# Monoamine oxidase B gene variants associated with attention deficit hyperactivity disorder in the Indo-Caucasoid population from West Bengal

**DOI:** 10.1186/s12863-016-0401-6

**Published:** 2016-06-24

**Authors:** Arijit Karmakar, Subhamita Maitra, Barnali Chakraborti, Deepak Verma, Swagata Sinha, Kochupurackal P. Mohanakumar, Usha Rajamma, Kanchan Mukhopadhyay

**Affiliations:** Manovikas Biomedical Research and Diagnostic Centre, 482, Madudah, Plot I-24, Sec.-J, E.M. Bypass, Kolkata, 700107 India; Indian Institute of Chemical Biology-Council of Scientific & Industrial Research, Jadavpur, Kolkata, 700 032 India

**Keywords:** ADHD, *MAOB*, rs56220155, Conduct problems, Linkage disequilibrium, Multifactor dimensionality reduction, Indo-Caucasoid population

## Abstract

**Background:**

Attention deficit hyperactivity disorder (ADHD) is characterized by symptoms of inattention, excessive motor activity and impulsivity detected mostly during childhood. These traits are known to be controlled by monoamine neurotransmitters, chiefly dopamine, serotonin and norepinephrine. Monoamine oxidase A (MAOA) and B (MAOB), two isoenzymes bound to the outer membrane of mitochondria, are involved in the degradation of monoamines and were explored for association with ADHD in different ethnic groups. In the present study, few exonic as well as intronic *MAOB* variants were analyzed in ADHD probands (*N* = 150) and ethnically matched controls (*N* = 150) recruited following the Diagnostic and Statistical Manual for Mental Disorders-4^th^ edition (DSM-IV). Appropriate scales were used for measuring the behavioural attributes. Gene variants were analyzed by amplification of target sites followed by DNA sequencing and data obtained were analyzed by population based statistical methods.

**Results:**

Out of 34 variants present in the analyzed sites, only seven functional variants, rs4824562, rs56220155, rs2283728, rs2283727, rs3027441, rs6324 and rs3027440, were found to be polymorphic. rs2283728 *‘C’* (*P* = 3.45e-006) and rs3027440 *‘T’* (*P* = 0.02) alleles showed higher frequencies in ADHD probands as compared to controls. rs56220155 *‘A’* (*P* = 0.04) allele and *‘GA’* (*P* = 0.04) genotype showed higher frequencies in the male and female ADHD probands respectively as compared to sex-matched controls. Analysis of pairwise linkage disequilibrium revealed striking differences between probands and controls. Haplotype analysis revealed significantly higher occurrence of different haplotypes in the ADHD probands while some haplotypes were detected in the controls only. Higher scores for conduct problems were found to be associated with rs56220155 *‘A’* (*P* = 0.05) allele in the male ADHD probands. Multifactor dimensionality reduction analysis showed independent as well as interactive effects of polymorphic variants which were more robust in the male probands.

**Conclusions:**

Since all the polymorphic variants analyzed were functional, it may be inferred that *MAOB* gene variants are contributing to the etiology of ADHD in the Indo-Caucasoid population from eastern India which merits further in depth analysis.

**Electronic supplementary material:**

The online version of this article (doi:10.1186/s12863-016-0401-6) contains supplementary material, which is available to authorized users.

## Background

Attention deficit hyperactivity disorder (ADHD) is an etiologically complex behavioural disorder. Major symptoms include persistent age-inappropriate hyperactivity and impulsivity, sometimes in association with inattention [1], leading to impairments in academic performances as well as social life [2, 3]. Worldwide ADHD is highly prevalent and boys are more prone to the disorder than girls [4, 5]. In India also, ADHD is quite prevalent and diagnosed more frequently in boys than girls [6–8]. Co-morbidity with other psychiatric disorders is common and in such condition, impairment is more [9, 10].

Being a multi-factorial genetic disorder with around 76 % heritability [11, 12], genetics is believed to play significant role in the etiology of ADHD [13, 14]. Neurotransmitters like dopamine, serotonin, and norepinephrine regulate all vital behavioural attributes and studies on candidate genes involved in the regulation of these neurotransmitters [15, 16] revealed associations between altered dopaminergic transmission and behavioural as well as cognitive deficits in various populations [17, 18].

Monoamine oxidase A (MAOA) and B (MAOB) are mitochondrial outer membrane bound isoenzymes, catalyzing oxidative deamination of neurotransmitters like dopamine, serotonin, norepinephrine, and other neuromodulators like benzylamine, phenylethylamine (PEA), tyramine and tryptamine in the brain as well as peripheral tissues [19, 20]. The two isoenzymes differ in substrate specificity [20, 21]; while MAOA preferentially oxidizes serotonin and norepinephrine, MAOB prefers benzylamine and PEA. MAOB activity in human increases with age [22] and is selectively inhibited by low concentration of deprenyl; but in high concentration, the selectivity is lost [23]. In the human brain, MAOB is the key enzyme degrading dopamine [24–26] and subcortical regions exhibit higher MAOB activity [22]. MAOB was hypothesized to control impulsivity, attention and vulnerability to ADHD by degrading dopamine [24, 25], which is the major factor responsible for regulating behaviour and cognitive function [18]. Further, *MAOB* knockout mice showed high level of PEA in the brain as well as an increased reactivity to stress and other behavioural alterations [20]. A correlation between platelet MAOB activity and sensation seeking as well as impulsiveness have also been reported [27, 28]. Platelet MAOB activity was used as a marker for psychic behaviour though it was not evident whether platelet MAOB activity was correlated with brain MAOB activity or not [29].

Several *MAOA* variants have showed association with ADHD in various populations, including the Indo-Caucasoid population [30, 31]. *MAOB* gene variants have also been studied, though the numbers of variants investigated were few and the data obtained were inconsistent [24, 28, 32–37]. Since genes encoding for MAOA and MAOB are located on the X-chromosome [24], we hypothesized that these genes may have a role in the sex bias of ADHD and our earlier study revealed a biased maternal transmission of *MAOA* variants to the male probands [30]. In this study, for the first time few *MAOB* variants were explored for association with ADHD and its associated phenotypic traits in the Indo-Caucasoid population.

## Methods

### Subject recruitment

Sample size of 150 was determined statistically [38] considering 8 % prevalence of ADHD in this population [6]. ADHD cases (126 males and 24 females) were recruited by child psychiatrist and clinical psychologist following the Diagnostic and Statistical Manual of Mental Disorders-4^th^ edition (DSM-IV) criteria [1]. 73.34 % of the recruited cases were of the combined subtype, while hyperactive-impulsive and inattentive subtypes were of 13.33 % each. Mean age of the ADHD cases was 7.69 ± 2.99 years (range 3 to 18 years). Psychological evaluation was done through - The revised Conners’ Parent Rating Scale (CPRS-R) [39] and Wechsler Intelligence Scale for Children >5 years [40]/Developmental Screening Test [41] for children < 5 years for the inattention-hyperactivity level and intelligent quotient (IQ) status respectively. DSM-IV score for oppositional defiant disorder (ODD) and Parental Account of Children’s Symptoms (PACS) score for conduct problems were also used for assessing the traits in ADHD probands. Patients with any other neuropsychiatric disorders, mental retardation (IQ ≤ 70) including Down syndrome and Fragile-X syndrome, pervasive developmental disorder were excluded from the study.

The control group comprised of 150 ethnically matched healthy individuals (125 males and 25 females) assessed by the same psychometric evaluation procedure. Mean age of the control individuals was 18.41 ± 8.78 years (range 3 to 28 years).

### Genotyping

Peripheral blood collected from the study participants was used for genomic DNA preparation using the standard protocol [42]. The target regions (detailed in Additional file 1) were amplified via polymerase chain reaction using primers (provided in Additional file 2) designed in the lab using the Primer3 software [43]. Applied Biosystems 3130 Genetic Analyzer with 98.5 % base calling accuracy and Read Length of upto 950 bp was used for sequence analysis of the amplicons using Big Dye v 3.1 chemistry and Sequencing Analysis Software, v 5.2 (Additional file 2). Chromatograms were also analyzed manually and mis-spaced letters/double peaks were investigated carefully for genotyping. For identification of heterozygous SNPs, >25 % base calling was accepted. Function of polymorphic variants was analyzed *in silico* using the is-rSNP [44].

### Comparison with other ethnic groups

Allelic and genotypic frequencies of African (ACB: African Caribbeans in Barbados; ASW: Americans of African Ancestry in SW USA; ESN: Esan in Nigeria; LWK: Luhya in Webuye, Kenya; MAG: Mandinka in The Gambia; MSL: Mende in Sierra Leone; YRI: Yoruba in Ibadan, Nigeria), American (CLM: Colombians from Medellin, Colombia; MXL: Mexican Ancestry from Los Angeles USA; PEL: Peruvians from Lima, Peru; PUR: Puerto Ricans from Puerto Rico), East Asian (CDX: Chinese Dai in Xishuangbanna, China; CHB: Han Chinese in Bejing, China; CHS: Southern Han Chinese; JPT: Japanese in Tokyo, Japan; KHW: Kinh in Ho Chi Minh City, Vietnam), European (CEU: Utah Residents (CEPH) with Northern and Western European Ancestry; FIN: Finnish in Finland; GBR: British in England and Scotland; IBS: Iberian Population in Spain; TSI: Toscani in Italia); South Asian (BEB: Bengali from Bangladesh; GIH: Gujarati Indian from Houston, Texas; ITU: Indian Telugu from the UK; PJL: Punjabi from Lahore, Pakistan; STU: Sri Lankan Tamil from the UK) ancestry were retrieved from the 1000 Genomes Project Phase 3 (32) database [45] and compared with that of the Indo-Caucasoid (IND) control population (natives of the eastern Indian state of West Bengal; 23°N, 87°E).

### Statistical analyses of data

To test the Hardy-Weinberg equilibrium [46], genotypic counts of only female ADHD probands and ethnically matched female controls were used since the *MAOB* gene is X-linked [24] and it is still unclear whether the male hemizygotes and female homozygotes are equivalent or not [47]. Allelic and genotypic association analyses for individual polymorphism as well as haplotype analysis were carried out using the UNPHASED v 3.1.5 [48] and correction for multiple testing was done while running the UNPHASED at 1000-fold iteration. To examine genotypic association, only female cases and female controls were considered. To calculate the power of the tests showing significant association, Piface version 1.72 [49] was used. Online odds ratio calculator [50] was used to calculate the odds ratio (OR). Pairwise linkage disequilibrium (LD) between the variants was measured using the Haploview program version 4.2 [51].

### Analysis of interaction between the variants

Interaction between the variants or epistasis was analyzed by the Multifactor Dimensionality Reduction (MDR) program [52] through a 4-step process using the case-control data set. In the first step, using filter methods, interesting polymorphisms were selected from the pool of possible candidates through entropy-based measures of information gain (IG) for each individual polymorphism/attribute (i.e. main effects) and each pairwise combination of attributes (i.e. two way interaction effects) [53]. In the second step, a new multilocus attribute, which capture interaction information is constructed using previously selected polymorphisms in conjunction with constructive induction algorithm. Thus multilocus genotypes were pooled into high-risk and low-risk groups, effectively reducing the dimensionality of the attributes from multiple dimensions to one dimension. In the third step, the new multilocus attribute constructed in the previous step was evaluated using a machine learning method (i.e. naive Bayes classifier, based on probability theory). In the final step*,* an interaction circle graph, using the entropy estimates from step 1, was depicted by the program. Interaction circle graph comprised of a node for each attribute (i.e. polymorphism) with pairwise connections between them. The percentage of entropy (i.e. information gain or IG) by each polymorphism was visualized on each node, while the IG for each pairwise combination of polymorphisms was visualized on each connection. Thus, the independent main effects of each polymorphism were quickly compared to the interaction effect between them. Positive entropy values indicated synergy, while negative entropy values indicated redundancy [53]. All these analyses were implemented in the open-source MDR software package version 2.0 beta 8.4.

### Association of alleles with phenotypic traits

Based on the CPRS-R, ‘T scores’, ranging between 38 and 90, were obtained for ADHD probands. DSM-IV scores (ranging between 0 and 36) for assessing ODD trait and PACS scores (ranging between 0 and 90) for assessing conduct problems were also obtained. Male probands were sub-grouped based on the presence/absence of the derived allele for each variant and distribution pattern of behavioural scores in each of the two comparing groups was checked using the Kolmogorov-Smirnov normality test [54]. Equality of variances was also checked using two sample F-test [55]. Allelic association with behavioural scores was analyzed using the Student’s t-test [56] only when the variables (i.e. behavioural scores) showed a normal distribution and variances were equal. In other conditions, nonparametric test such as Mann-Whitney test [56] was performed. As the number of female probands was limited, comparative analysis on behavioural scores and genotypes was not performed for this group.

## Results

Out of 34 variants localized in the investigated regions, only seven, rs4824562, rs56220155, rs2283728, rs2283727, rs3027441, rs6324 and rs3027440, were found to be polymorphic in the studied population (Additional file 1). *In silico* analysis revealed that all these variants have potential regulatory function (Additional file 3).

Frequencies of rs4824562 *‘G’,* rs3027441*‘C’*, rs6324 *‘T’* and rs3027440 *‘C’* alleles in the IND population revealed significant differences as compared to several world populations, while the distribution pattern of rs4824562, rs6324 and rs3027440 matched with populations from the South Asia (Table 1). On the other hand, rs56220155 exhibited statistically significant difference even with populations from South Asia (Table 1, BEB and GIH, *P* < 0.05), chiefly due to an increase in the minor allele frequency in the IND. rs2283727 also exhibited significant differences in the derived *‘A’* allele frequency as compared to all other populations except BEB, ITU, PJL, STU with South Asian ancestry (Table 1). No information was available in the 1000 Genomes Project Phase 3 (32) database for rs2283728.

**Table 1:** Comparative analysis on allelic and genotypic frequencies in different populations [1000 Genomes Project Phase 3 (32)]

Variants with respective alleles	Super-populations	Populations	Allele count (frequency)		Chi-square (*p*-value)	Genotype count (frequency)			Chi-square (*p*-value)
			*1*	*2*		*1/1*	*1/2*	*2/2*	
rs4824562 [*A (1), G (2)*]	AFR	ACB	140 (0.97)	5 (0.03)	27.80 (**0.00**)	46 (0.94)	3 (0.06)	0 (0.00)	16.30 (**0.00**)
		ASW	93 (0.97)	3 (0.03)	20.20 (**0.00**)	32 (0.91)	3 (0.09)	0 (0.00)	11.00 (**0.00**)
		ESN	144 (0.99)	1 (0.01)	38.10 (**0.00**)	45 (0.98)	1 (0.02)	0 (0.00)	20.30 (**0.00**)
		LWK	153 (0.99)	1 (0.01)	40.50 (**0.00**)	54 (0.98)	1 (0.02)	0 (0.00)	24.10 (**0.00**)
		MAG	170 (0.99)	1 (0.01)	44.80 (**0.00**)	57 (0.98)	1 (0.02)	0 (0.00)	25.40 (**0.00**)
		MSL	127 (0.99)	1 (0.01)	33.70 (**0.00**)	43 (1.00)	0 (0.00)	0 (0.00)	22.60 (**0.00**)
		YRI	163 (0.99)	1 (0.01)	43.00 (**0.00**)	56 (1.00)	0 (0.00)	0 (0.00)	28.50 (**0.00**)
	AMR	CLM	110 (0.76)	35 (0.24)	0.01 (0.93)	25 (0.49)	22 (0.43)	4 (0.08)	1.00 (0.61)
		MXL	79 (0.82)	17 (0.18)	1.69 (0.19)	17 (0.53)	13 (0.41)	2 (0.06)	0.83 (0.66)
		PEL	85 (0.66)	44 (0.34)	3.31 (0.07)	16 (0.36)	25 (0.57)	3 (0.07)	3.96 (0.14)
		PUR	135 (0.88)	19 (0.12)	8.02 (**0.01**)	35 (0.70)	14 (0.28)	1 (0.02)	3.72 (0.16)
	EAS	CDX	112 (0.79)	30 (0.21)	0.53 (0.47)	28 (0.57)	19 (0.39)	2 (0.04)	1.75 (0.42)
		CHB	134 (0.84)	26 (0.16)	3.54 (0.06)	39 (0.68)	14 (0.25)	4 (0.07)	1.28 (0.53)
		CHS	141 (0.89)	17 (0.11)	10.70 (**0.00**)	40 (0.75)	13 (0.25)	0 (0.00)	7.64 (**0.02**)
		JPT	130 (0.86)	22 (0.14)	5.21 (**0.02**)	33 (0.69)	14 (0.29)	1 (0.02)	3.41 (0.18)
		KHV	128 (0.84)	24 (0.16)	3.85 (**0.05**)	41 (0.77)	10 (0.19)	2 (0.04)	4.16 (0.13)
	EUR	CEU	125 (0.84)	24 (0.16)	3.51 (0.06)	35 (0.70)	13 (0.26)	2 (0.04)	2.31 (0.31)
		FIN	131 (0.82)	29 (0.18)	2.06 (0.15)	41 (0.67)	18 (0.30)	2 (0.03)	2.70 (0.26)
		GBR	116 (0.85)	20 (0.15)	4.61 (**0.03**)	32 (0.71)	12 (0.27)	1 (0.02)	3.41 (0.18)
		IBS	142 (0.89)	18 (0.11)	9.96 (**0.00**)	39 (0.74)	13 (0.25)	1 (0.01)	4.51 (0.11)
		TSI	135 (0.84)	26 (0.16)	3.65 (0.06)	41 (0.76)	11 (0.20)	2 (0.04)	3.79 (0.15)
	SAS	BEB	98 (0.75)	32 (0.25)	0.00 (0.99)	27 (0.62)	16 (0.36)	1 (0.02)	2.77 (0.25)
		GIH	110 (0.73)	40 (0.27)	0.19 (0.67)	29 (0.62)	13 (0.28)	5 (0.10)	0.22 (0.90)
		ITU	115 (0.79)	30 (0.21)	0.68 (0.41)	25 (0.58)	16 (0.37)	2 (0.05)	1.30 (0.52)
		PJL	116 (0.81)	28 (0.19)	1.20 (0.27)	29 (0.60)	18 (0.38)	1 (0.02)	3.14 (0.21)
		STU	119 (0.80)	30 (0.20)	0.91 (0.34)	29 (0.62)	14 (0.30)	4 (0.08)	0.32 (0.85)
		IND	132 (0.75)	43 (0.25)	-	14 (0.56)	8 (0.32)	3 (0.12)	-
rs56220155 [*G (1), A (2)*]	AFR	ACB	58 (0.40)	87 (0.60)	0.27 (0.60)	6 (0.12)	30 (0.61)	13 (0.27)	7.77 (**0.02**)
		ASW	37 (0.39)	59 (0.61)	0.05 (0.82)	2 (0.06)	18 (0.51)	15 (0.43)	3.99 (0.14)
		ESN	55 (0.38)	90 (0.62)	0.02 (0.89)	8 (0.17)	18 (0.39)	20 (0.44)	1.12 (0.57)
		LWK	59 (0.38)	95 (0.62)	0.05 (0.83)	11 (0.20)	23 (0.42)	21 (0.38)	2.27 (0.32)
		MAG	68 (0.40)	103 (0.60)	0.25 (0.62)	9 (0.16)	29 (0.50)	20 (0.34)	3.93 (0.14)
		MSL	61 (0.48)	67 (0.52)	3.36 (0.07)	8 (0.19)	27 (0.62)	8 (0.19)	10.70 (**0.01**)
		YRI	69 (0.42)	95 (0.58)	0.86 (0.35)	10 (0.17)	25 (0.45)	21 (0.38)	2.62 (0.27)
	AMR	CLM	20 (0.14)	125 (0.86)	22.20 (**0.00**)	2 (0.04)	10 (0.20)	39 (0.76)	4.64 (0.10)
		MXL	10 (0.10)	86 (0.90)	22.10 (**0.00**)	1 (0.03)	3 (0.09)	28 (0.88)	7.32 (**0.03**)
		PEL	7 (0.05)	122 (0.95)	41.30 (**0.00**)	0 (0.00)	6 (0.14)	38 (0.86)	10.70 (**0.01**)
		PUR	53 (0.34)	101 (0.66)	0.27 (0.61)	7 (0.14)	24 (0.48)	19 (0.38)	2.89 (0.24)
	EAS	CDX	16 (0.11)	126 (0.89)	27.60 (**0.00**)	1 (0.02)	7 (0.14)	41 (0.84)	8.13 (**0.02**)
		CHB	26 (0.16)	134 (0.84)	18.40 (**0.00**)	1 (0.02)	15 (0.26)	41 (0.72)	6.46 (**0.04**)
		CHS	23 (0.15)	135 (0.85)	21.80 (**0.00**)	0 (0.00)	17 (0.32)	36 (0.68)	8.95 (**0.01**)
		JPT	21 (0.14)	131 (0.86)	22.80 (**0.00**)	1 (0.02)	10 (0.21)	37 (0.77)	6.06 (**0.05**)
		KHV	15 (0.10)	137 (0.90)	32.70 (**0.00**)	1 (0.02)	10 (0.19)	42 (0.79)	7.21 (**0.03**)
	EUR	CEU	35 (0.23)	114 (0.77)	7.03 (**0.01**)	3 (0.06)	16 (0.32)	31 (0.62)	1.97 (0.38)
		FIN	45 (0.28)	115 (0.72)	3.08 (0.08)	5 (0.08)	26 (0.43)	30 (0.49)	2.18 (0.34)
		GBR	45 (0.33)	91 (0.67)	0.55 (0.46)	4 (0.09)	23 (0.51)	18 (0.40)	3.61 (0.16)
		IBS	49 (0.31)	111 (0.69)	1.58 (0.21)	4 (0.08)	26 (0.49)	23 (0.43)	3.53 (0.17)
		TSI	44 (0.27)	117 (0.73)	3.68 (0.06)	6 (0.11)	19 (0.35)	29 (0.54)	0.61 (0.74)
	SAS	BEB	33 (0.25)	97 (0.75)	4.73 (**0.03**)	1 (0.02)	22 (0.50)	21 (0.48)	6.20 (**0.05**)
		GIH	30 (0.20)	120 (0.80)	11.50 (**0.00**)	3 (0.06)	15 (0.32)	29 (0.62)	1.72 (0.42)
		ITU	54 (0.37)	91 (0.63)	0.00 (0.99)	7 (0.16)	17 (0.40)	19 (0.44)	1.05 (0.59)
		PJL	46 (0.32)	98 (0.68)	0.94 (0.33)	3 (0.06)	20 (0.42)	25 (0.52)	2.51 (0.29)
		STU	48 (0.32)	101 (0.68)	0.86 (0.35)	1 (0.02)	26 (0.55)	20 (0.43)	7.80 (**0.02**)
		IND	65 (0.37)	110 (0.63)	-	4 (0.16)	7 (0.28)	14 (0.56)	-
rs2283727 [*C (1), A (2)*]	AFR	ACB	132 (0.91)	13 (0.09)	14.20 (**0.00**)	40 (0.82)	9 (0.18)	0 (0.00)	7.63 (**0.02**)
		ASW	84 (0.88)	12 (0.12)	6.04 (**0.01**)	27 (0.77)	8 (0.23)	0 (0.00)	4.97 (0.08)
		ESN	124 (0.86)	21 (0.14)	5.57 (**0.02**)	35 (0.76)	10 (0.22)	1 (0.02)	3.64 (0.16)
		LWK	140 (0.91)	14 (0.09)	14.50 (**0.00**)	45 (0.82)	8 (0.15)	2 (0.03)	4.67 (0.10)
		MAG	155 (0.91)	16 (0.09)	15.00 (**0.00**)	50 (0.86)	7 (0.12)	1 (0.02)	7.99 (**0.02**)
		MSL	112 (0.88)	16 (0.12)	7.44 (**0.01**)	33 (0.77)	9 (0.21)	1 (0.02)	3.48 (0.18)
		YRI	143 (0.87)	21 (0.13)	8.32 (**0.00**)	47 (0.84)	8 (0.14)	1 (0.02)	6.70 (**0.04**)
	AMR	CLM	145 (1.00)	0 (0.00)	42.30 (**0.00**)	51 (1.00)	0 (0.00)	0 (0.00)	23.50 (**0.00**)
		MXL	96 (1.00)	0 (0.00)	28.80 (**0.00**)	32 (1.00)	0 (0.00)	0 (0.00)	15.50 (**0.00**)
		PEL	127 (0.98)	2 (0.02)	32.20 (**0.00**)	42 (0.95)	2 (0.05)	0 (0.00)	14.40 (**0.00**)
		PUR	149 (0.97)	5 (0.03)	31.00 (**0.00**)	48 (0.96)	2 (0.04)	0 (0.00)	16.60 (**0.00**)
	EAS	CDX	130 (0.91)	12 (0.09)	15.00 (**0.00**)	43 (0.88)	5 (0.10)	1 (0.02)	7.90 (**0.02**)
		CHB	141 (0.88)	19 (0.12)	9.64 (**0.00**)	43 (0.75)	13 (0.23)	1 (0.02)	4.52 (0.10)
		CHS	138 (0.87)	20 (0.13)	8.34 (**0.00**)	39 (0.74)	14 (0.26)	0 (0.00)	6.83 (**0.03**)
		JPT	131 (0.86)	21 (0.14)	6.55 (**0.01**)	37 (0.77)	10 (0.21)	1 (0.02)	3.99 (0.14)
		KHV	140 (0.92)	12 (0.08)	17.10 (**0.00**)	44 (0.83)	9 (0.17)	0 (0.00)	8.56 (**0.01**)
	EUR	CEU	148 (0.99)	1 (0.01)	40.30 (**0.00**)	49 (0.98)	1 (0.02)	0 (0.00)	19.40 (**0.00**)
		FIN	160 (1.00)	0 (0.00)	46.30 (**0.00**)	61 (1.00)	0 (0.00)	0 (0.00)	27.60 (**0.00**)
		GBR	134 (0.99)	2 (0.01)	34.00 (**0.00**)	44 (0.98)	1 (0.02)	0 (0.00)	17.50 (**0.00**)
		IBS	160 (1.00)	0 (0.00)	46.30 (**0.00**)	53 (1.00)	0 (0.00)	0 (0.00)	24.30 (**0.00**)
		TSI	161 (1.00)	0 (0.00)	46.60 (**0.00**)	54 (1.00)	0 (0.00)	0 (0.00)	24.70 (**0.00**)
	SAS	BEB	103 (0.79)	27 (0.21)	0.80 (0.37)	26 (0.59)	18 (0.41)	0 (0.00)	6.02 (**0.05**)
		GIH	127 (0.85)	23 (0.15)	4.75 (**0.03**)	32 (0.68)	12 (0.26)	3 (0.06)	0.82 (0.66)
		ITU	101 (0.70)	44 (0.30)	1.08 (0.30)	21 (0.49)	17 (0.40)	5 (0.11)	0.97 (0.62)
		PJL	109 (0.76)	35 (0.24)	0.03 (0.86)	31 (0.65)	14 (0.29)	3 (0.06)	0.72 (0.70)
		STU	119 (0.80)	30 (0.20)	1.15 (0.28)	30 (0.64)	17 (0.36)	0 (0.00)	6.01 (**0.05**)
		IND	131 (0.75)	44 (0.25)	-	15 (0.60)	7 (0.28)	3 (0.12)	-
rs3027441 [*C (1), T (2)*]	AFR	ACB	25 (0.17)	120 (0.83)	0.77 (0.38)	1 (0.02)	16 (0.33)	32 (0.65)	1.89 (0.39)
		ASW	18 (0.19)	78 (0.81)	0.22 (0.64)	0 (0.00)	13 (0.37)	22 (0.63)	3.65 (0.16)
		ESN	31 (0.21)	114 (0.79)	0.00 (0.96)	3 (0.06)	15 (0.33)	28 (0.61)	0.59 (0.75)
		LWK	16 (0.10)	138 (0.90)	7.01 (**0.01**)	2 (0.04)	10 (0.18)	43 (0.78)	1.18 (0.55)
		MAG	22 (0.13)	149 (0.87)	4.19 (**0.04**)	1 (0.02)	13 (0.22)	44 (0.76)	2.07 (0.36)
		MSL	21 (0.16)	107 (0.84)	1.07 (0.30)	1 (0.02)	12 (0.28)	30 (0.70)	1.25 (0.54)
		YRI	29 (0.18)	135 (0.82)	0.65 (0.42)	3 (0.05)	10 (0.18)	43 (0.77)	0.71 (0.70)
	AMR	CLM	12 (0.08)	133 (0.92)	10.10 (**0.00**)	0 (0.00)	7 (0.14)	44 (0.86)	5.81 (0.06)
		MXL	7 (0.07)	89 (0.93)	8.75 (**0.00**)	0 (0.00)	3 (0.09)	29 (0.91)	5.35 (0.07)
		PEL	22 (0.17)	107 (0.83)	0.79 (0.37)	2 (0.04)	13 (0.30)	29 (0.66)	0.52 (0.77)
		PUR	11 (0.07)	143 (0.93)	12.9 (**0.00**)	0 (0.00)	5 (0.10)	45 (0.90)	7.20 (**0.03**)
	EAS	CDX	17 (0.12)	125 (0.88)	4.67 (**0.03**)	1 (0.02)	9 (0.18)	39 (0.80)	2.00 (0.37)
		CHB	27 (0.17)	133 (0.83)	0.99 (0.32)	1 (0.02)	17 (0.30)	39 (0.68)	2.06 (0.36)
		CHS	32 (0.20)	126 (0.80)	0.04 (0.84)	1 (0.02)	19 (0.36)	33 (0.62)	2.48 (0.29)
		JPT	37 (0.24)	115 (0.76)	0.48 (0.50)	2 (0.04)	18 (0.38)	28 (0.58)	1.60 (0.45)
		KHV	16 (0.10)	136 (0.90)	6.75 (**0.01**)	1 (0.02)	9 (0.17)	43 (0.81)	2.47 (0.29)
	EUR	CEU	1 (0.01)	148 (0.99)	32.60 (**0.00**)	0 (0.00)	1 (0.02)	49 (0.98)	14.30 (**0.00**)
		FIN	0 (0.00)	160 (1.00)	38.00 (**0.00**)	0 (0.00)	0 (0.00)	61 (1.00)	21.50 (**0.00**)
		GBR	2 (0.01)	134 (0.99)	27.00 (**0.00**)	0 (0.00)	1 (0.02)	44 (0.98)	12.90 (**0.00**)
		IBS	0 (0.00)	160 (1.00)	38.00 (**0.00**)	0 (0.00)	0 (0.00)	53 (1.00)	18.90 (**0.00**)
		TSI	0 (0.00)	161 (1.00)	38.30 (**0.00**)	0 (0.00)	0 (0.00)	54 (1.00)	19.20 (**0.00**)
	SAS	BEB	27 (0.21)	103 (0.79)	0.01 (0.94)	0 (0.00)	18 (0.41)	26 (0.59)	5.03 (0.08)
		GIH	23 (0.15)	127 (0.85)	1.81 (0.18)	3 (0.06)	12 (0.26)	32 (0.68)	0.08 (0.96)
		ITU	44 (0.30)	101 (0.70)	3.55 (0.06)	5 (0.11)	17 (0.40)	21 (0.49)	2.37 (0.31)
		PJL	35 (0.24)	109 (0.76)	0.45 (0.50)	3 (0.06)	14 (0.29)	31 (0.65)	0.26 (0.88)
		STU	30 (0.20)	119 (0.80)	0.05 (0.82)	0 (0.00)	17 (0.36)	30 (0.64)	4.56 (0.10)
		IND	37 (0.21)	138 (0.79)	-	2 (0.08)	6 (0.24)	17 (0.68)	-
rs6324 [*C (1), T (2)*]	AFR	ACB	141 (0.97)	4 (0.03)	24.00 (**0.00**)	46 (0.94)	3 (0.06)	0 (0.00)	9.57 (**0.01**)
		ASW	92 (0.96)	4 (0.04)	13.90 (**0.00**)	32 (0.91)	3 (0.09)	0 (0.00)	6.09 (**0.05**)
		ESN	135 (0.93)	10 (0.07)	12.80 (**0.00**)	42 (0.91)	3 (0.07)	1 (0.02)	6.26 (**0.04**)
		LWK	149 (0.97)	5 (0.03)	23.60 (**0.00**)	51 (0.93)	3 (0.05)	1 (0.02)	8.24 (**0.02**)
		MAG	165 (0.96)	6 (0.04)	24.70 (**0.00**)	54 (0.93)	4 (0.07)	0 (0.00)	10.20 (**0.01**)
		MSL	123 (0.96)	5 (0.04)	18.40 (**0.00**)	38 (0.88)	5 (0.12)	0 (0.00)	5.75 (0.06)
		YRI	155 (0.95)	9 (0.05)	17.70 (**0.00**)	52 (0.93)	3 (0.05)	1 (0.02)	8.46 (**0.02**)
	AMR	CLM	134 (0.92)	11 (0.08)	11.40 (**0.00**)	45 (0.88)	6 (0.12)	0 (0.00)	6.51 (**0.04**)
		MXL	89 (0.93)	7 (0.07)	8.75 (**0.00**)	29 (0.91)	3 (0.09)	0 (0.00)	5.35 (0.07)
		PEL	108 (0.84)	21 (0.16)	1.14 (0.29)	30 (0.68)	12 (0.27)	2 (0.05)	0.39 (0.82)
		PUR	146 (0.95)	8 (0.05)	17.60 (**0.00**)	46 (0.92)	4 (0.08)	0 (0.00)	8.34 (**0.02**)
	EAS	CDX	125 (0.88)	17 (0.12)	4.67 (**0.03**)	39 (0.80)	9 (0.18)	1 (0.02)	2.00 (0.37)
		CHB	133 (0.83)	27 (0.17)	0.99 (0.32)	39 (0.68)	17 (0.30)	1 (0.02)	2.06 (0.36)
		CHS	126 (0.80)	32 (0.20)	0.04 (0.84)	33 (0.62)	19 (0.36)	1 (0.02)	2.48 (0.29)
		JPT	115 (0.76)	37 (0.24)	0.48 (0.49)	28 (0.58)	18 (0.38)	2 (0.04)	1.60 (0.45)
		KHV	136 (0.90)	16 (0.10)	6.75 (**0.01**)	43 (0.81)	9 (0.17)	1 (0.02)	2.47 (0.29)
	EUR	CEU	148 (0.99)	1 (0.01)	32.60 (**0.00**)	49 (0.98)	1 (0.02)	0 (0.00)	14.30 (**0.00**)
		FIN	160 (1.00)	0 (0.00)	38.00 (**0.00**)	61 (1.00)	0 (0.00)	0 (0.00)	21.50 (**0.00**)
		GBR	134 (0.99)	2 (0.01)	27.00 (**0.00**)	44 (0.98)	1 (0.02)	0 (0.00)	12.90 (**0.00**)
		IBS	160 (1.00)	0 (0.00)	38.00 (**0.00**)	53 (1.00)	0 (0.00)	0 (0.00)	18.90 (**0.00**)
		TSI	161 (1.00)	0 (0.00)	38.30 (**0.00**)	54 (1.00)	0 (0.00)	0 (0.00)	19.20 (**0.00**)
	SAS	BEB	103 (0.79)	27 (0.21)	0.01 (0.94)	26 (0.59)	18 (0.41)	0 (0.00)	5.03 (0.08)
		GIH	127 (0.85)	23 (0.15)	1.81 (0.18)	32 (0.68)	12 (0.26)	3 (0.06)	0.08 (0.96)
		ITU	101 (0.70)	44 (0.30)	3.55 (0.06)	21 (0.49)	17 (0.40)	5 (0.11)	2.37 (0.31)
		PJL	109 (0.76)	35 (0.24)	0.45 (0.50)	31 (0.65)	14 (0.29)	3 (0.06)	0.26 (0.88)
		STU	119 (0.80)	30 (0.20)	0.05 (0.82)	30 (0.64)	17 (0.36)	0 (0.00)	4.56 (0.10)
		IND	138 (0.79)	37 (0.21)	-	17 (0.68)	6 (0.24)	2 (0.08)	-
rs3027440 [*T (1), C (2)*]	AFR	ACB	141 (0.97)	4 (0.03)	34.40 (**0.00**)	46 (0.94)	3 (0.06)	0 (0.00)	7.79 (**0.02**)
		ASW	92 (0.96)	4 (0.04)	20.90 (**0.00**)	32 (0.91)	3 (0.09)	0 (0.00)	4.89 (0.09)
		ESN	135 (0.93)	10 (0.07)	21.60 (**0.00**)	42 (0.91)	3 (0.07)	1 (0.02)	4.63 (0.10)
		LWK	149 (0.97)	5 (0.03)	34.30 (**0.00**)	51 (0.93)	3 (0.05)	1 (0.02)	6.24 (**0.04**)
		MAG	165 (0.97)	6 (0.03)	36.30 (**0.00**)	54 (0.93)	4 (0.07)	0 (0.00)	8.30 (**0.02**)
		MSL	123 (0.96)	5 (0.04)	27.40 (**0.00**)	38 (0.88)	5 (0.12)	0 (0.00)	4.71 (0.10)
		YRI	155 (0.95)	9 (0.05)	28.00 (**0.00**)	52 (0.93)	3 (0.05)	1 (0.02)	6.42 (**0.04**)
	AMR	CLM	134 (0.92)	11 (0.08)	19.80 (**0.00**)	45 (0.88)	6 (0.12)	0 (0.00)	5.40 (0.07)
		MXL	89 (0.93)	7 (0.07)	14.90 (**0.00**)	29 (0.91)	3 (0.09)	0 (0.00)	4.28 (0.12)
		PEL	108 (0.84)	21 (0.16)	4.79 (**0.03**)	30 (0.68)	12 (0.27)	2 (0.05)	0.70 (0.70)
		PUR	146 (0.95)	8 (0.05)	27.60 (**0.00**)	46 (0.92)	4 (0.08)	0 (0.00)	6.78 (**0.03**)
	EAS	CDX	125 (0.88)	17 (0.12)	10.80 (**0.00**)	39 (0.80)	9 (0.18)	1 (0.02)	1.60 (0.45)
		CHB	133 (0.83)	27 (0.17)	4.84 (**0.03**)	39 (0.68)	17 (0.30)	1 (0.02)	2.51 (0.29)
		CHS	126 (0.80)	32 (0.20)	2.00 (0.16)	33 (0.62)	19 (0.36)	1 (0.02)	3.28 (0.19)
		JPT	115 (0.76)	37 (0.24)	0.27 (0.60)	28 (0.58)	18 (0.38)	2 (0.04)	2.53 (0.28)
		KHV	136 (0.90)	16 (0.10)	13.9 (**0.00**)	43 (0.81)	9 (0.17)	1 (0.02)	1.92 (0.38)
	EUR	CEU	148 (0.99)	1 (0.01)	43.70 (**0.00**)	49 (0.98)	1 (0.02)	0 (0.00)	12.00 (**0.00**)
		FIN	160 (1.00)	0 (0.00)	50.00 (**0.00**)	61 (1.00)	0 (0.00)	0 (0.00)	18.60 (**0.00**)
		GBR	134 (0.98)	2 (0.02)	37.20 (**0.00**)	44 (0.98)	1 (0.02)	0 (0.00)	10.70 (**0.01**)
		IBS	160 (1.00)	0 (0.00)	50.00 (**0.00**)	53 (1.00)	0 (0.00)	0 (0.00)	16.30 (**0.00**)
		TSI	161 (1.00)	0 (0.00)	50.30 (**0.00**)	54 (1.00)	0 (0.00)	0 (0.00)	16.60 (**0.00**)
	SAS	BEB	103 (0.79)	27 (0.21)	1.50 (0.22)	26 (0.59)	18 (0.41)	0 (0.00)	6.03 (**0.05**)
		GIH	127 (0.85)	23 (0.15)	6.35 (**0.01**)	32 (0.68)	12 (0.26)	3 (0.06)	0.31 (0.86)
		ITU	101 (0.70)	44 (0.30)	0.47 (0.49)	21 (0.49)	17 (0.40)	5 (0.11)	3.55 (0.17)
		PJL	109 (0.76)	35 (0.24)	0.27 (0.60)	31 (0.65)	14 (0.29)	3 (0.06)	0.74 (0.69)
		STU	119 (0.80)	30 (0.20)	2.01 (0.16)	30 (0.64)	17 (0.36)	0 (0.00)	5.32 (0.07)
		IND	128 (0.73)	47 (0.27)	-	18 (0.72)	5 (0.20)	2 (0.08)	-

*AFR* African, *ACB* African Caribbeans in Barbados, *ASW* Americans of African Ancestry in SW USA, *ESN* Esan in Nigeria, *LWK* Luhya in Webuye, Kenya, *MAG* Mandinka in The Gambia, *MSL* Mende in Sierra Leone, *YRI* Yoruba in Ibadan, Nigeria, *AMR* American, *CLM*, Colombians from Medellin, Colombia, *MXL* Mexican Ancestry from Los Angeles USA, *PEL* Peruvians from Lima, Peru, *PUR* Puerto Ricans from Puerto Rico, *EAS* East Asian, *CDX* Chinese Dai in Xishuangbanna, China, *CHB* Han Chinese in Bejing, China, *CHS* Southern Han Chinese, *JPT* Japanese in Tokyo, Japan, *KHW* Kinh in Ho Chi Minh City, Vietnam, *EUR* European, *CEU* Utah Residents (CEPH) with Northern and Western European Ancestry, *FIN* Finnish in Finland, *GBR* British in England and Scotland, *IBS* Iberian Population in Spain, *TSI* Toscani in Italia, *SAS* South Asian, *BEB* Bengali from Bangladesh, *GIH* Gujarati Indian from Houston, Texas, *ITU* Indian Telugu from the UK, *PJL* Punjabi from Lahore, Pakistan, *STU* Sri Lankan Tamil from the UK, *IND* Indo-Caucasoid control population. Significant differences are presented in bold

Case-control comparative analysis revealed statistically significant higher occurrence of rs2283728 *‘C’* (*P* = 1.21e-005; power = 99 %) and rs3027440 *‘T’* (*P* = 0.04; power = 53 %) alleles in the probands (Table 2, Additional file 4). Stratification based on gender revealed statistically significant higher occurrence of rs2283728 *‘C’* (*P* = 3.45e-006; power = 99.63 %), rs3027440 *‘T’* (*P* = 0.02; power = 66 %) and rs56220155 *‘A’* (*P* = 0.04; power = 54 %) alleles in the male probands in comparison to the male controls (Table 2, Additional file 4). All the variants followed the HWE in the female subjects (Additional file 5). Statistically significant higher occurrence of rs56220155 *‘GA’* genotype (*P* = 0.04; OR = 3.92; 95 % confidence interval (CI) = 1.28–11.95; power = 62 %) was also observed in the female probands as compared to the female controls (Additional file 5). Rest of the investigated variants did not show any biased occurrence (Additional files 4 and 5).

**Table 2:** Comparative analysis on allelic frequencies of *MAOB* variants showing significant association with ADHD

Variants	Alleles	Groups	Control	Proband	Chi-square (*p*-value)	Odds Ratio (95 % confidence interval)	Power
rs56220155	*G*	Male^a^	0.4	0.28	4.2 (0.04)	1.72 (1.02–2.9)	54 %
	*A*		0.6	0.72			
rs2283728	*T*	All^b^	0.42	0.21	19.15 (1.21e-005)	2.71 (1.73–4.26)	99 %
	*C*		0.58	0.79			
	*T*	Male^a^	0.47	0.2	21.55 (3.45e-006)	3.4 (2.01–5.74)	99.63 %
	*C*		0.53	0.8			
rs3027440	*T*	All^b^	0.73	0.82	4.13 (0.04)	1.68 (1.02–2.78)	53 %
	*C*		0.27	0.18			
	*T*	Male^a^	0.7	0.83	5.83 (0.02)	2.03 (1.14–3.62)	66 %
	*C*		0.3	0.17			

^a^N = 125 Control/126 proband

^b^N = 125 male and 25 female controls/126 male and 24 female probands

**Fig. 1:**
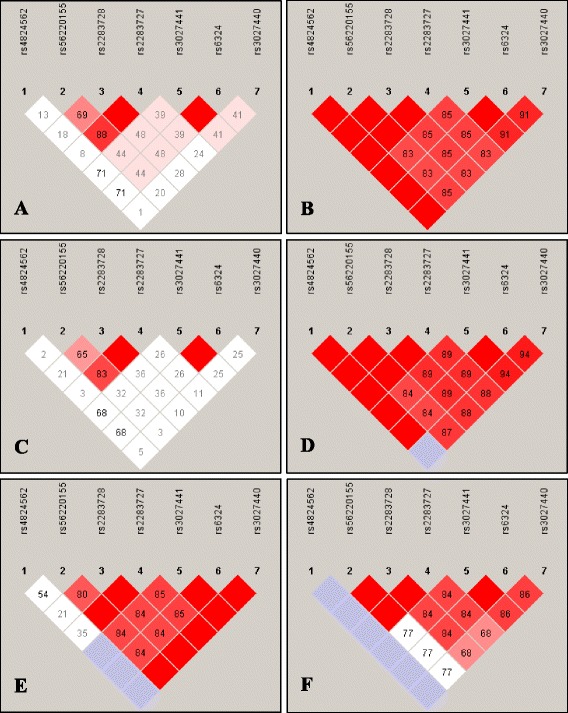
Pairwise linkage disequilibrium between the studied variants. **a** controls; **b** ADHD probands; **c** male controls; **d** male probands; **e** female controls; **f** female probands. *D’* is a measure of frequency of association of alleles at 2 loci and numbers represent the *D’* value expressed as a percentile. Diamonds without numbers represent *D’* values of 1.0

Pairwise LD analyses showed strikingly varied patterns (Fig. 1, Additional file 6); LD of the ADHD group was significantly different from that of the control group (Fig. 1a, b). Subjects stratified on the basis of gender revealed significant difference between the male subjects and sex-matched controls (Fig. 1c, d). Striking differences in the pattern of the LDs were also noticed between female ADHD probands and female control group (Fig. 1e, f).

The *C-T* haplotype, formed between rs2283728 and rs3027440 having strong LD (D’ 0.84, r^2^ 0.58), exhibited significant higher occurrence (*P* = 1.07e-008; OR = 3.6) in the probands (Table 3). Significant higher occurrence of the *A-C-T* haplotype of rs56220155-rs2283728-rs3027440 (*P* = 1.99e-009; OR = 4.52) was also noticed in the male probands (Table 3). Haplotype analysis for all the seven variants (rs4824562-rs56220155-rs2283728-rs2283727-rs3027441-rs6324-rs3027440) showed statistically significant higher occurrence of four haplotypes (*A-A-C-C-T-C-T*, *A-G-C-C-T-C-T*, *A-G-T-A-C-T-C* and *G-A-C-C-T-C-T*) in the probands (*P* ≤ 0.04; OR > 2) (Table 3). Stratified analysis based on gender, also revealed statistically significant higher occurrence of three haplotypes (*A-A-C-C-T-C-T*, *A-G-T-A-C-T-C* and *G-A-C-C-T-C-T*) in the male probands (*P* ≤ 0.005; OR > 3) and nominally significant higher occurrence of the *A-G-C-C-T-C-T* haplotype in the female probands (*P* = 0.055; OR = 8.23) as compared to sex-matched controls (Table 3).

**Table 3:** Population-based comparative analysis on haplotype frequency

Groups	Variant combinations	`Haplotypes	Control	Proband	Chi-square (*p*-value)	Odds Ratio (95 % confidence interval)
All^a^	rs4824562-rs56220155-rs2283728-rs2283727-rs3027441-rs6324-rs3027440	*A-A-C-C-T-C-C*	0.04	0	5.27 (**0.02**)	0.13 (0.03–0.65)
		*A-A-C-C-T-C-T*	0.32	0.49	12.14 (**0.0005**)	2.08 (1.36–3.19)
		*A-A-T-C-T-C-C*	0.02	0	4.02 (**0.04**)	0.13 (0.02–0.96)
		*A-A-T-C-T-C-T*	0.03	0	6.07 (**0.01**)	0.13 (0.03–0.66)
		*A-G-C-C-T-C-T*	0.03	0.09	4.06 (**0.04**)	2.5 (1.04–6.03)
		*A-G-T-A-C-T-C*	0.07	0.15	4.29 (**0.038**)	2.21 (1.13–4.32)
		*A-G-T-C-T-C-T*	0.03	0	5.04 (**0.02**)	0.13 (0.02–0.78)
		*G-A-C-C-T-C-C*	0.03	0	4.03 (**0.04**)	0.13 (0.02–0.88)
		*G-A-C-C-T-C-T*	0.08	0.18	6.26 (**0.01**)	2.46 (1.33–4.58)
		*G-A-T-C-T-C-T*	0.02	0	4.02 (**0.04**)	0.13 (0.02–0.96)
		*G-G-T-A-T-C-C*	0.02	0	4.06 (**0.04**)	0.13 (0.02–0.91)
		*G-G-T-A-T-C-T*	0.04	0	7.05 (**0.008**)	0.13 (0.03–0.57)
		*G-G-T-C-T-C-T*	0.02	0	3.01 (0.08)	0.14 (0.01–1.3)
	rs2283728-rs3027440	*C-C*	0.11	0.02	9.43 (**0.002**)	0.25 (0.11–0.57)
		*C-T*	0.47	0.77	32.72 (**1.07e-008**)	3.6 (2.34–5.55)
		*T-C*	0.16	0.16	0.07 (0.78)	0.98 (0.55–1.75)
		*T-T*	0.27	0.05	28.27 (**1.05e-007**)	0.2 (0.12–0.36)
Male^b^	rs4824562-rs56220155-rs2283728-rs2283727-rs3027441-rs6324-rs3027440	*A-A-C-C-T-C-C*	0.05	0	6.2 (**0.01**)	0.13 (0.03–0.65)
		*A-A-C-C-T-C-T*	0.26	0.53	19.98 (**7.85e-006**)	3.16 (1.2–5.23)
		*A-A-T-C-T-C-C*	0.03	0	4.1 (**0.04**)	0.13 (0.02–0.94)
		*A-A-T-C-T-C-T*	0.03	0	4.1 (**0.04**)	0.13 (0.02–0.94)
		*A-G-T-A-C-T-C*	0.04	0.15	8.91 (**0.003**)	3.58 (1.55–8.3)
		*A-G-T-A-T-C-T*	0.06	0.008	5.71 (**0.016**)	0.2 (0.05–0.75)
		*A-G-T-C-T-C-T*	0.04	0	5.14 (**0.02**)	0.13 (0.02–0.76)
		*G-A-C-C-T-C-C*	0.03	0	4.1 (**0.04**)	0.13 (0.02–0.94)
		*G-A-C-C-T-C-T*	0.06	0.17	7.75 (**0.005**)	3.04 (1.39–6.66)
		*G-A-T-C-T-C-T*	0.03	0	4.1 (**0.04**)	0.13 (0.02–0.94)
		*G-G-T-A-T-C-C*	0.03	0	4.1 (**0.04**)	0.13 (0.02–0.94)
		*G-G-T-A-T-C-T*	0.02	0	3.06 (0.08)	0.13 (0.01–1.28)
		*G-G-T-C-T-C-T*	0.02	0	3.06 (0.08)	0.13 (0.01–1.28)
	rs56220155-rs2283728-rs3027440	*A-C-C*	0.13	0.02	11.85 (**0.0006**)	0.19 (0.07–0.49)
		*A-C-T*	0.33	0.71	35.98 (**1.99e-009**)	4.52 (2.76–7.42)
		*A-T-C*	0.05	0	6.2 (**0.01**)	0.13 (0.03–0.65)
		*A-T-T*	0.1	0	12.7 (**0.0004**)	0.12 (0.04–0.39)
		*G-C-C*	0.02	0	2.03 (0.15)	0.13 (0.008–2.14)
		*G-C-T*	0.06	0.08	0.54 (0.46)	1.45 (0.54–3.86)
		*G-T-C*	0.11	0.16	1.17 (0.28)	1.49 (0.72–3.06)
		*G-T-T*	0.22	0.04	17.54 (**2.82e-005**)	0.21 (0.1–0.43)
Female^c^	rs4824562-rs56220155-rs2283728-rs2283727-rs3027441-rs6324-rs3027440	*A-G-C-C-T-C-T*	0	0.08	3.69 (**0.055**)	8.23 (1.13–59.77)
		*G-G-T-A-T-C-T*	0.08	0	4.16 (**0.04**)	0.13 (0.02–0.97)

^a^ *N* = 125 male and 25 female controls/126 male and 24 female probands

^b^ *N* = control 125/126 probands

^c^ *N* = 25 control/24 probands

Statistically significant differences are presented in bold

**Fig. 2:**
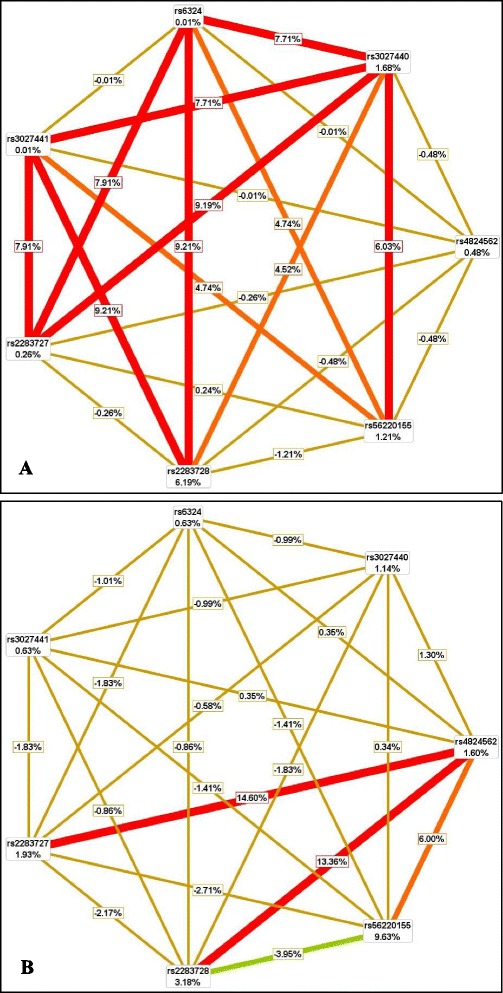
Interaction graph generated through Multifactor Dimensionality Reduction (MDR) software. **a** Population based analysis for male subjects; **b** Population based analysis for female subjects. The graphical interaction model describe the percentage of entropy (i.e. information gain or IG) in case-control status that is explained by each factor (i.e. gene variant) or two-way interaction. Two-way interactions between factors are depicted by line accompanied by a percent of entropy explained by that interaction. Values inside large boxes on nodes indicate information gain (IG) of individual/independent main effect of each polymorphic variant, whereas values inside small boxes between nodes exemplify IG of pairwise combination/interactive effects of respective variants. Positive IG values, between the nodes, indicate the synergistic interactions; whereas negative IG values indicate the redundancy between the respective nodes/variants. Schematic coloration represents a continuum from synergy to redundancy. The red lines represent a high degree of synergy. The orange lines represent moderate synergy. The golden yellow lines accompanied by a positive percent of entropy represent minimal synergy, whereas the golden yellow lines accompanied by a negative percent of entropy represent minimal redundancy. Green line represents high redundancy

MDR analysis using data of male controls and ADHD probands revealed strong pairwise interactions between different variants (Fig. 2a, Additional file 7). rs2283728 showed highest independent main effect (nodal IG = 6.19 %) followed by rs3027440 (nodal IG = 1.68 %) and rs56220155 (nodal IG = 1.21 %). rs3027440 showed high degree of synergistic interactive effects with rs56220155, rs2283727, rs3027441, rs6324 and moderate degree of synergistic interactive effect with rs2283728 (Fig. 2a, Additional file 7). rs6324 and rs3027441 separately interacted with rs2283728 and rs2283727 in pairwise combinations showing high degree of synergy alongside rs3027440, and interacted with rs56220155 showing moderate degree of synergy (Fig. 2a, Additional file 7). A nominal synergy was observed between rs56220155 and rs2283727 (Fig. 2a, Additional file 7). rs4824562 showed minimum redundant effects with individual polymorphic variants (Fig. 2a, Additional file 7). Minimal redundancies were also observed between rs56220155 and rs2283728, rs2283728 and rs2283727, rs3027441 and rs6324 respectively (Fig. 2a, Additional file 7).

Stratified analysis for female subjects showed highest independent main effect of rs56220155 (nodal IG = 9.63 %) followed by rs2283728, rs2283727, rs4824562, and rs3027440 (nodal IG = 3.18 %, 1.93 %, 1.60 %, and 1.14 % respectively). High degree of synergistic effects of rs4824562 with rs2283728 and rs2283727, along with moderate synergistic effect with rs56220155, and minimal synergistic effect with the rest of the variants were also noticed (Fig. 2b, Additional file 8). rs56220155, showing moderate degree of synergy with rs4824562, also showed minimal synergy with rs3027440, minimal redundancy with rs2283727, rs3027441, rs6324, and highest redundancy with rs2283728 by pairwise combinations (Fig. 2b, Additional file 8). rs2283728, rs2283727, rs3027441, rs6324 and rs3027440 showed minimal redundancy with each other in pairwise combinations (Fig. 2b, Additional file 8).

CPRS-R ‘T scores’ for oppositional behaviour [62.07 ± 15.81], cognitive problems/inattention [73.02 ± 9.82], hyperactivity [73.95 ± 12.10], and ADHD index [71.63 ± 8.25] confirmed the disease associated traits. DSM-IV scores for ODD trait [13.81 ± 8.19] and PACS scores for conduct problems [17.34 ± 11.68] were also noticeable. Male ADHD probands harbouring the rs56220155 *‘A’* allele showed significantly higher mean score for conduct problems as compared to the those having the *‘G’* allele (Table 4). DSM-IV scores for ODD trait and CPRS-R ‘T scores’ failed to show any significant association with any allele (Additional file 9).

**Table 4:** Association of alleles with PACS scores of male ADHD probands (*N* = 126)

Variants	Alleles	PACS scores for conduct problems	
		Mean ± SE	*p*-value^a^
rs4824562	*A*	18.19 ± 1.69	0.37
	*G*	16.75 ± 4.19	
rs56220155	*G*	14.42 ± 2.42	**0.05**
	*A*	19.81 ± 1.94	
rs2283728	*T*	14.46 ± 2.73	0.11
	*C*	19.05 ± 1.83	
rs2283727	*C*	19.05 ± 1.83	0.11
	*A*	14.46 ± 2.73	
rs3027441	*C*	16 ± 2.99	0.27
	*T*	18.47 ± 1.79	
rs6324	*C*	18.47 ± 1.79	0.27
	*T*	16 ± 2.99	
rs3027440	*T*	18.6 ± 1.74	0.19
	*C*	14.78 ± 3.18	

^a^Analyzed through Student’s t-test; statistically significant differences are presented in bold

## Discussion

MAOB is a key enzyme in the human brain, modulating oxidation of dopamine [24–26] as well as benzylamine, PEA, tyramine, and tryptamine [19–21]. Previous genetic association studies on *MAOB* revealed inconsistent findings in different populations. To find out the role of *MAOB* variants in the etiology of eastern Indian ADHD probands, we used a four step approach. Initially, allelic and genotypic frequencies of *MAOB* variants were analyzed by population-based methods to identify risk variants in the ADHD probands. Then, LD between the studied variants was analyzed to understand whether these variants are working independently or in a pairwise clubbed manner. Next, we verified independent main effects and epistatic effects of *MAOB* variants using case-control data set. Finally, to identify the relevance of these gene variants in disease associated symptoms, we analyzed association between alleles and behavioural attributes.

Alleles/genotypes of the eastern Indian control population studied in the present investigation resembled the South Asian ancestral population for most of the variants. However, allelic frequencies of rs2283727 and rs3027440 differed from the Gujrati population while rs56220155 differed from both Bengali from Bangladesh and Gujrati Indian from Houston, principally due to an increase in the minor allele frequency. We have earlier reported vast difference in the frequency of alleles in the Indian population [57]. Whether this drift in allelic frequencies is conferring any specific advantage is a matter of conjecture at the moment and merits further investigation in a large number of samples belonging to each ethnic group.

Earlier investigators reported significant positive association of *MAOB* gene variants with ADHD in the Spanish probands [37], while in the Irish [33] and Czech [28] population no association was noticed. International Multi-centre ADHD Gene project, with Caucasian subjects from 12 specialized centres in eight different countries, also failed to notice any association between ADHD *and MAOB* gene variants, including rs4824562, rs6324 and rs3027440 [34]. In the present investigation, out of 34 variants only 7 were identified to be polymorphic and *in silico* analysis revealed that all can potentially regulate *MAOB* transcription. Four variants, rs56220155, rs2283728, rs2283727 and rs3027441, were analyzed for the first time for association with ADHD. Variants like rs4824562 and rs3027440 were previously studied in the European Caucasoid probands [34], while rs6324 was studied in ADHD probands belonging to Han Chinese [24, 35] as well as European Caucasoid [34] populations; in the Han Chinese population, significant positive [24] as well as negative associations [35] were reported for rs6324. This site failed to show any positive association with ADHD in the Indo-Caucasoid population.

rs2283728 and rs3027440 showed allelic as well as haplotypic associations with ADHD in the Indo-Caucasoid population. rs56220155 showed genotypic association in the female probands. All these three variants also showed association in the male ADHD probands. Haplotypes consisting of all the seven variants, including the above three, showed significant association with the disorder. It can be hypothesized from the present observation that these three variants independently as well as in combinations may play an important role in ADHD.

We have noticed a striking difference in the LD pattern of ADHD probands and controls; pairwise all variants were in strong LD in the ADHD probands as compared to the controls. In absence of ethnic differences, recruitment of related individuals, and consanguineous marriage, the observed difference in the LD pattern may suggest a lower rate of recombination between the studied variants in the probands which facilitates generation of risk haplotypes associated with the disease etiology. Epistasis analysis showed significant pairwise synergistic interactive effects of most of the variants in the male ADHD probands. In female probands the interactive effects were very less. However, the number of female probands was limited and further exploration on the matter is desired before reaching into any conclusion. Allele *‘A’* of rs56220155 was associated with high conduct problems, as measured by the PACS score, in the male ADHD probands. The *‘A’* allele of rs56220155 also showed statistically significant higher occurrence in the male ADHD probands in comparison to the male controls. It can be inferred from these observations that *MAOB* has a significant role in the etiology of ADHD.

## Conclusions

Our investigation for the first time revealed association of rs56220155 and rs2283728 with ADHD. rs3027440, previously reported to have no association in the Euro-Caucasoid ADHD subjects [34], also revealed positive association in the Indo-Caucasoid population. rs2283727 and rs3027441, in strong LD with rs2283728 and rs6324 respectively, were investigated for the first time in ADHD probands and statistical analysis failed to show any association in the studied population. The observed difference in allelic/genotypic association in the present study could be attributed to difference in allelic frequencies since the IND population revealed a different allelic distribution pattern as compared to other ancestral ethnic groups from other parts of the world. Stratified analysis revealed gross difference in the LD pattern of male and female ADHD probands as compared to sex-matched controls possibly be due to absence of recombination between the sites in the probands, thus creating a block conferring risk of ADHD. We have also noticed higher frequencies of rs56220155 *‘A’*, rs2283728 “*C*” and rs3027440 “*T*” alleles in the male probands. Male probands exhibiting conduct problems also showed higher frequency of rs56220155 *‘A’*. Whether this is really a male specific effect is a matter of conjecture at the moment since the major limitation of the present study was the low number of female subjects investigated. The high odd’s ratio (>2) observed for a few association analyses could also be attributed to the limitation in sample number. We may infer from the data obtained that further investigation on a large cohort of samples belonging to different ethnic groups is warranted to validate our observation in the Indo-Caucasoid population from the eastern India.

## Abbreviations

ACB, African Caribbeans in Barbados; ADHD, attention deficit hyperactivity disorder; AFR, African; AMR, American; ASW, Americans of African Ancestry in SW USA; BEB, Bengali from Bangladesh; CDX, Chinese Dai in Xishuangbanna, China; CEU, Utah Residents (CEPH) with Northern and Western European Ancestry; CHB, Han Chinese in Bejing, China; CHS, Southern Han Chinese; CI, confidence interval; CLM, Colombians from Medellin, Colombia; CPRS-R, Conners’ Parent Rating Scale-revised; DNA, deoxyribonucleic acid; DSM-IV, Diagnostic and Statistical Manual of Mental Disorders-4^th^ edition; EAS, East Asian; ESN, Esan in Nigeria; EUR, European; FIN, finnish in Finland; GBR, British in England and Scotland; GIH, Gujarati Indian from Houston, Texas; HWE, Hardy-Weinberg equilibrium; IBS, Iberian Population in Spain; IG, information gain; IND, Indo-Caucasoid control population; IQ, intelligent quotient; is-rSNP, *in silico* regulatory single nucleotide polymorphism; ITU, Indian Telugu from the UK; JPT, Japanese in Tokyo, Japan; KHW, Kinh in Ho Chi Minh City, Vietnam; LD, Linkage disequilibrium; LWK, Luhya in Webuye, Kenya; MAG, Mandinka in The Gambia; MAOA, Monoamine oxidase A; MAOB, Monoamine oxidase B; MDR, Multifactor Dimensionality Reduction; MSL, Mende in Sierra Leone; MXL, Mexican Ancestry from Los Angeles USA; ODD, oppositional defiant disorder; OR, odds ratio; PACS, Parental Account of Children’s Symptoms; PEA, phenylethylamine; PEL, Peruvians from Lima, Peru; PJL, Punjabi from Lahore, Pakistan; PUR, Puerto Ricans from Puerto Rico; SAS, South Asian; STU, Sri Lankan Tamil from the UK; TSI, Toscani in Italia; YRI, Yoruba in Ibadan, Nigeria

## Additional files

Additional file 1: Details of the *MAOB* variants investigated. Description: The table summarizes details of the *MAOB* variants investigated. (XLSX 11 kb) Additional file 2: Protocol followed for analysis of *MAOB* target regions. Description: The table summarizes the protocol followed for analysis of target sites. (XLSX 8 kb) Additional file 3: Functional significance of *MAOB* polymorphic variants predicted *in silico* using is-rSNP. Description: The table summarizes functional regulatory role of *MAOB* polymorphic variants. (XLSX 40 kb) Additional file 4: Population-based comparative analysis on allelic frequencies of *MAOB* variants. Description: The table summarizes allelic frequencies of *MAOB* variants in ADHD cases and ethnically matched controls. (XLSX 10 kb) Additional file 5: Genotypic distribution of *MAOB* variants in female subjects. Description: The table summarizes comparative analysis on genotypic distribution of *MAOB* variants in female ADHD cases and sex matched controls. (XLSX 10 kb) Additional file 6: Pairwise Linkage Disequilibrium pattern of *MAOB* variants (analyzed using Haploview 4.2). Description: The table summarizes the *D’* and *r* ^*2*^ values for pairwise LD analysis in ADHD probands and ethnically matched controls. (XLSX 11 kb) Additional file 7: MDR analysis of case-control data set of male subjects. Description: The table summarizes independent as well as interactive effects of *MAOB* variants in case-control status for male subjects. (XLSX 8 kb) Additional file 8: MDR analysis of case-control data set of female subjects. Description: The table summarizes the independent as well as interactive effects of *MAOB* variants in case-control status of female subjects. (XLSX 9 kb) Additional file 9: Analysis of allelic association with DSM-IV scores for ODD trait and CPRS-R ‘T scores’ for oppositional behavior, cognitive problems/inattention, hyperactivity, and ADHD index in male ADHD probands. Description: This table summarizes statistical comparisons between the mean scores and alleles. (XLSX 10 kb)
